# Application of ethanol alleviates heat damage to leaf growth and yield in tomato

**DOI:** 10.3389/fpls.2024.1325365

**Published:** 2024-02-19

**Authors:** Daisuke Todaka, Do Thi Nhu Quynh, Maho Tanaka, Yoshinori Utsumi, Chikako Utsumi, Akihiro Ezoe, Satoshi Takahashi, Junko Ishida, Miyako Kusano, Makoto Kobayashi, Kazuki Saito, Atsushi J. Nagano, Yoshimi Nakano, Nobutaka Mitsuda, Sumire Fujiwara, Motoaki Seki

**Affiliations:** ^1^ Plant Genomic Network Research Team, RIKEN Center for Sustainable Resource Science, Yokohama, Kanagawa, Japan; ^2^ Agricultural Genetics Institute, Hanoi, Vietnam; ^3^ Plant Epigenome Regulation Laboratory, RIKEN Cluster for Pioneering Research, Wako, Saitama, Japan; ^4^ Metabolomics Research Group, RIKEN Center for Sustainable Resource Science, Yokohama, Kanagawa, Japan; ^5^ Graduate School of Life and Environmental Sciences, University of Tsukuba, Tsukuba, Ibaraki, Japan; ^6^ Tsukuba Plant Innovation Research Center, University of Tsukuba, Tsukuba, Ibaraki, Japan; ^7^ Faculty of Agriculture, Ryukoku University, Otsu, Shiga, Japan; ^8^ Institute for Advanced Biosciences, Keio University, Yamagata, Japan; ^9^ Bioproduction Research Institute, National Institute of Advanced Industrial Science and Technology (AIST), Tsukuba, Japan; ^10^ Kihara Institute for Biological Research, Yokohama City University, Yokohama, Kanagawa, Japan; ^11^ Graduate School of Science and Engineering, Saitama University, Saitama, Saitama, Japan

**Keywords:** ethanol, heat stress, chemical priming, micro-tom, transcriptome, metabolome

## Abstract

Chemical priming has emerged as a promising area in agricultural research. Our previous studies have demonstrated that pretreatment with a low concentration of ethanol enhances abiotic stress tolerance in Arabidopsis and cassava. Here, we show that ethanol treatment induces heat stress tolerance in tomato (*Solanum lycopersicon* L.) plants. Seedlings of the tomato cultivar ‘Micro-Tom’ were pretreated with ethanol solution and then subjected to heat stress. The survival rates of the ethanol-pretreated plants were significantly higher than those of the water-treated control plants. Similarly, the fruit numbers of the ethanol-pretreated plants were greater than those of the water-treated ones. Transcriptome analysis identified sets of genes that were differentially expressed in shoots and roots of seedlings and in mature green fruits of ethanol-pretreated plants compared with those in water-treated plants. Gene ontology analysis using these genes showed that stress-related gene ontology terms were found in the set of ethanol-induced genes. Metabolome analysis revealed that the contents of a wide range of metabolites differed between water- and ethanol-treated samples. They included sugars such as trehalose, sucrose, glucose, and fructose. From our results, we speculate that ethanol-induced heat stress tolerance in tomato is mainly the result of increased expression of stress-related genes encoding late embryogenesis abundant (LEA) proteins, reactive oxygen species (ROS) elimination enzymes, and activated gluconeogenesis. Our results will be useful for establishing ethanol-based chemical priming technology to reduce heat stress damage in crops, especially in Solanaceae.

## 1 Introduction

Heat stress is one of the most serious problems in agriculture. A recent report by Intergovernmental Panel on Climate Change (IPCC) 2022 shows that the average global surface air temperature in 2030s will be 1.5°C higher than the average in 1850-1900 (Pörtner et al., 2022). The frequency of heat stress conditions has increased with global climate change. Heat stress decreases crop productivity, leading to negative impacts on society and the economy. Therefore, enhancing the heat stress tolerance of crops is an important goal for plant researchers.

Chemical priming is one of the useful techniques that can increase the stress tolerance of plants (Savvides et al., 2016; Sako et al., 2021b). A wide variety of chemical compounds can elicit molecular mechanisms governing environmental stress tolerance (Savvides et al., 2016; Sako et al., 2021b; Zulfiqar et al., 2022). In the practical use of chemical priming in agriculture, it is important to optimize the method for each situation. For different crop species, for example, the application of different concentrations of the chemical priming compound will be critical for achieving the desired physiological traits.

Recent studies have confirmed that application of ethanol to plants can enhance environmental stress tolerance. We previously showed that ethanol treatment enhanced heat stress tolerance in Arabidopsis through the activated unfolded protein response (UPR) mechanism (Matsui et al., 2022). Ethanol-treated Arabidopsis plants also showed enhanced drought tolerance through activated stomatal closure and gluconeogenesis (Bashir et al., 2022). In addition, ethanol treatment enhanced drought tolerance in wheat and rice (Bashir et al., 2022), soybean (Rahman et al., 2022) and cassava (Vu et al., 2022), salt stress tolerance in Arabidopsis and rice (Nguyen et al., 2017) and soybean (Das et al., 2022), and high-light stress tolerance in Arabidopsis (Sako et al., 2021a). Ethanol is readily available and is considered to be environment- and human-friendly. From these perspectives, it is expected that farmers might prefer ethanol chemical priming over other chemical options, and ultimately social acceptance of the method is likely.

Heat stress damages plant growth and yield (Hoshikawa et al., 2021; Ahmad et al., 2022). Extensive research has elucidated how photosynthesis is highly sensitive to heat stress (Hu et al., 2020). The damaged photosynthetic processes include electron transport, CO_2_ assimilation, chlorophyll biosynthesis, and thylakoid membrane fluidity (Hu et al., 2020). Impaired photosynthesis leads to growth retardation. Heat stress also causes oxidative damage by reactive oxygen species (ROS) such as superoxide radical, singlet oxygen, hydroxyl radical and hydrogen peroxide (Fortunato et al., 2023). To cope with ROS molecules, organisms activate specific enzymes that reduce or inactivate ROS, including peroxidase, ascorbate peroxidase, glutathione reductase, superoxide dismutase, and catalase. Under severe heat stress conditions, proteins are denatured and functionally damaged. Heat shock proteins (HSPs) are induced by high temperatures and protect against protein denaturation through transcriptional cascades (Ohama et al., 2017).

Tomato (*Solanum lycopersicum* L.) is one of the most valuable vegetable crops because the fruit contains important nutrients for humans, such as vitamin A, vitamin C and lycopene. Tomato is also a representative species in the Solanaceae family. The plant has been used extensively in research. In particular, the model tomato cultivar ‘Micro-Tom’ has been widely studied. The superiority of ‘Micro-Tom’ is evident in its short generation time, small genome size, and stable genetic modification (Meissner et al., 1997). Genome-wide full-length cDNAs (Aoki et al., 2010) and the complete genome sequence (Kobayashi et al., 2014) of ‘Micro-Tom’ are available.

In the case of tomato, there have been no previous reports that ethanol priming can improve plant growth performance or survival under environmental stress conditions. In addition, little is known about the effects of ethanol treatment on fruit quality in crops. In this paper, we investigated whether ethanol application enhances heat stress tolerance in tomato ‘Micro-Tom’. We show that ethanol pretreatment alleviated heat damage to both vegetative growth and reproductive development. Transcriptome analysis identified the genes differentially expressed between water- and ethanol-treated seedlings and fruits. Furthermore, metabolome analysis unraveled the dynamics of changes in metabolites following ethanol application. Our results give new insight into ethanol-mediated heat stress tolerance in tomato and improve the understanding of such mechanisms more widely in plants.

## 2 Materials and methods

### 2.1 Plant materials and growth conditions

Seeds of tomato (*Solanum lycopersicum* L.) cultivar ‘Micro-Tom’ were obtained from the National Bioresource Project (MEXT, Japan) through the TOMATOMA database (Saito et al., 2011). Tomato seeds were imbibed in tap water at 22°C overnight under dark conditions. The imbibed seeds were sown into pots (70 mm diameter, 60 mm height, Yamato Plastic Co., Ltd., Nara, Japan) containing water-retaining horticultural clay granules (Seramis, Westland Horticulture Ltd. Tyrone, UK). After sowing, the pots were placed in a growth room set at 22°C with a 16-h light (110 to 140 µmol m^−2^ s^−1^ photosynthetic photon flux density)/8-h dark cycle.

### 2.2 Ethanol pretreatment and heat stress treatment

Sixteen-day-old plants grown in pots were used for ethanol pretreatment. The bottoms of the pots were placed in ethanol solution for 3 d. We selected 20 mM concentration of ethanol in pretreatment solution. This is based on the result of Arabidopsis and lettuce plants (Matsui et al., 2022). After ethanol pretreatment, the pots were placed in an air incubator (Sanyo incubator MIR 153, Sanyo Electric Co. Ltd, Osaka, Japan) set at 50 °C and maintained for the indicated time periods as heat stress treatment. During the heat treatment, the pots were electrically rotated in the air incubator to avoid location effects. After the heat treatment, the pots were returned to the growth room and maintained for the indicated time periods.

### 2.3 Estimation of green leaf areas

Green leaf areas were estimated by OpenCV (version 4.0.1) on Python 3.8.5. Photographs were taken above each plant. The hue, saturation, and value (HSV) color threshold range was set from [31, 70, 10] to [95, 255, 255].

### 2.4 Quantitative PCR

Six biological replicates for each treatment were used. One replicate of shoot or root samples consisted of three leaves or one root from one plant, respectively. Samples were put into a 10-mL tube, frozen in liquid nitrogen, and pulverized using a Multi-Beads Shocker system (Yasui Kikai, Osaka, Japan). Total RNA extraction, cDNA synthesis and PCR were performed as previously described (Matsui et al., 2022). Primer sequences are shown in **Supplementary Table S1**.

### 2.5 Transcriptome analysis

For the RNA-seq analysis, the sequencing library was prepared using the Lasy-Seq method (Kamitani et al., 2019). Specifically, 200 ng of total RNA was used per sample. The library was sequenced using the 151-bp paired-end mode of the HiSeq X Ten (Illumina, San Diego, CA, USA). RNA-seq analyses were performed with R1 reads. Low-quality reads and adapters were trimmed using Trimmomatic version 0.39 (http://www.usadellab.org/cms/?page=trimmomatic) with settings ‘ILLUMINACLIP : TruSeaq3-SE.fa:2:30:10 LEADING:3 TRAILING:3 SLIDINGWINDOW:4:15 MINLEN:36’. HISAT2 (http://daehwankim-lab.github.io/hisat2/) version 2.2.1 was used to map the reads to the *Solanum lycopersicum* SL4.0 reference genome with the ‘–max-intronlen 5000’ option. Aligned reads within gene models were counted using featureCounts version 2.0.1 (http://subread.sourceforge.net/) with the ‘–fracOverlap 0.5 -O -t gene -g ID -s 1 –primary’ options. Differentially expressed genes were identified using R version 4.0.4 (https://www.r-project.org/) and DESeq2 version 1.30.1 (https://bioconductor.org/packages/release/bioc/html/DESeq2.html) package. Genes with false discovery rate < 0.05 in each comparison were identified as differentially expressed. Gene ontology enrichment analysis was performed on the web tool (https://www.geneontology.org/).

### 2.6 Metabolome analysis by gas chromatography–time of flight/mass spectrometry

GC–TOF/MS analysis was carried out using the procedures described previously (Jonsson et al., 2005; Kusano et al., 2007a; Kusano et al., 2007b; Redestig et al., 2009) with slight modifications. Approximately 20 mg fresh weight of tissue per mL extraction medium containing ten stable isotope reference compounds was used in the extraction of metabolites.

## 3 Results

### 3.1 Effects of ethanol pretreatment on heat stress tolerance in tomato seedlings

Previously, we found that pretreatment with high concentrations of ethanol decreased shoot growth in Arabidopsis (Matsui et al., 2022). Therefore, we checked whether the 20 mM concentration of ethanol adversely affected shoot growth in tomato. The green leaf area of seedlings pretreated with 20 mM ethanol for 3 d and 6 d were not significantly different from those of the control seedlings pretreated with water (**Supplementary Figure S1**). These results suggested that 20 mM ethanol treatment did not inhibit shoot growth in tomato and so we proceeded to use that concentration in ethanol pretreatments of tomato thereafter.

**Figure 1:**
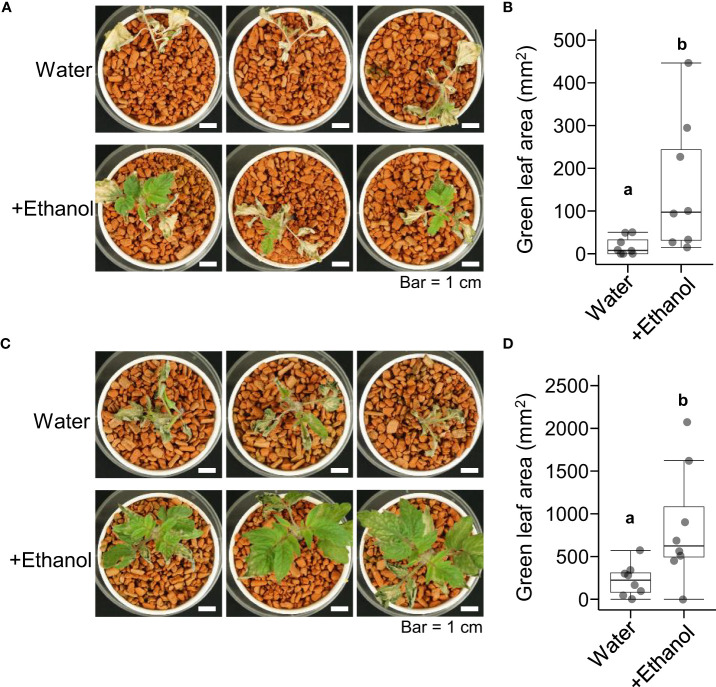
Heat tolerance of tomato ‘Micro-Tom’ seedlings treated with water or ethanol. Sixteen-day-old seedlings were pretreated with water or 20 mM ethanol for 3 d or 6 d then subjected to heat stress treatment (50 °C, 4 h). After the heat treatment, the seedlings were grown under normal conditions for 7 d. **(A)** Appearance of seedlings with 3 d pretreatment. **(B)** Box plot of green leaf areas of seedlings in **(A)**. **(C)** Appearance of seedlings with 6 d pretreatment. **(D)** Box plot of green leaf areas of seedlings in **(C)**. **(B, D)** n = 8. Different letters indicate significant differences at *P* < 0.05 (*t*-test).

Next, we investigated the effects of ethanol pretreatment on heat stress tolerance in tomato seedlings. After ethanol pretreatment for 3 d or 6 d, the seedlings were subjected to heat stress treatment of 50°C for 4 h then grown under normal growth conditions for 7 d. The green leaf areas of seedlings pretreated with ethanol for 3 d were greater than those of seedlings pretreated with water (**Figures 1A, B**). A similar result was observed in the seedlings of ethanol pretreatment for 6 d (**Figures 1C, D**). These results suggested that ethanol pretreatment reduced the leaf growth damage caused by heat stress in tomato seedlings.

### 3.2 Effects of ethanol pretreatment and heat stress treatment on tomato fruit development

**Figure 2:**
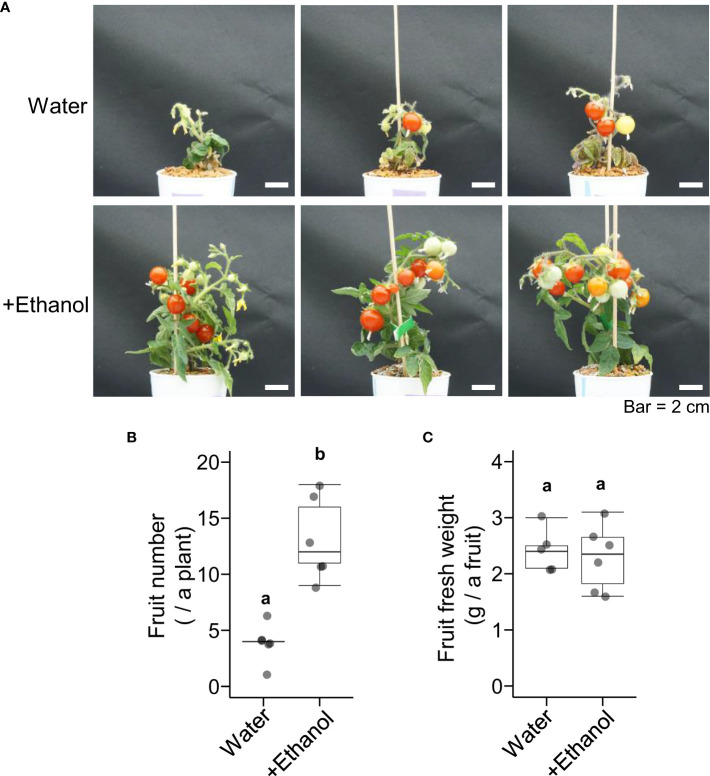
Effects of heat stress after ethanol treatment on tomato ‘Micro-Tom’ fruit development. Sixteen-day-old seedlings were pretreated with water or 20 mM ethanol for 3 d then subjected to heat stress treatment (50 °C, 2.5 h). After the heat treatment, the seedlings were grown under normal condition for 110 d. **(A)** Appearance of plants. **(B)** Box plot of fruit number. **(C)** Box plot of fruit fresh weight. **(B, C)** n = 5 to 6. Different letters indicate significant differences at *P* < 0.05 (*t*-test).

To investigate the effects of ethanol pretreatment and heat stress treatment on tomato fruit development, plants were grown until the mature fruit stage after ethanol pretreatment and heat stress treatment (**Figure 2A**). The appearance of plants after 1 d and 41 d of the heat treatment (seedling stage and flower developmental stage) was also shown in **Supplementary Figure S2**. The fruit numbers of the plants treated with ethanol were higher than those of the plants treated with water (**Figure 2B**). The fresh weight per fruit was not different between ethanol- and water-treated plants (**Figure 2C**). These data suggested that ethanol pretreatment might be effective for alleviating heat damage to fruit development.

### 3.3 Effects of ethanol pretreatment and heat stress treatment on water use efficiency

WUE is an important parameter that shows a relationship between plant growth and water use. To investigate the effects of ethanol pretreatment and heat stress treatment on WUE, we first estimated transpiration volumes during those periods. The transpiration volume during ethanol pretreatment was lower than that during water pretreatment (**Supplementary Figure S3A**). Next we checked transpiration volumes and leaf areas before heat stress treatment and after 9 d of heat stress treatment. During 9 d after the heat stress treatment, the transpiration volume and leaf area of ethanol-pretreated seedling were greater than those of water-pretreated seedling (**Supplementary Figures S3B, C**). Using these data, we calculated WUE during 9 d after the heat stress treatment by the formula: difference in green leaf area/transpiration volume. The result showed that the WUE of ethanol-pretreated seedling was not different from that of water-pretreated seedling (**Supplementary Figure S3D**). Finally, it was shown that the fresh weight of shoot of ethanol-pretreated seedling was higher than that of water-pretreated seedling (**Supplementary Figure S3E**).

### 3.4 Effects of ethanol pretreatment and heat stress treatment on plant biomass

Next we investigated the effects of ethanol pretreatment and heat stress treatment on plant biomass. The fresh and dry weights of shoots of seedlings pretreated with water were significantly decreased by heat stress treatment (**Supplementary Figures S4A, C**). On the other hand, the fresh and dry weights of shoots of seedlings pretreated with ethanol were not significantly decreased by the same stress treatment (**Supplementary Figures S4A, C**). These results suggest that ethanol pretreatment alleviated the shoot growth damage. In the case of roots, a similar result was acquired in the samples of dry weight, not in the samples of fresh weight (**Supplementary Figures S4B, D**).

### 3.5 Effects of ethanol pretreatment on stomatal aperture in tomato

Previous studies showed that ethanol treatment caused stomatal closing in Arabidopsis (Bashir et al., 2022) and cassava (Vu et al., 2022). We checked stomatal apertures in tomato plants treated with ethanol or water. As expected, the stomatal apertures of the seedlings treated with ethanol were smaller than those of the seedlings treated with water (**Supplementary Figure S5**).

### 3.6 Temporal and spatial gene expression profiles of ethanol-pretreated and heat stress-treated tomato plants

**Figure 3:**
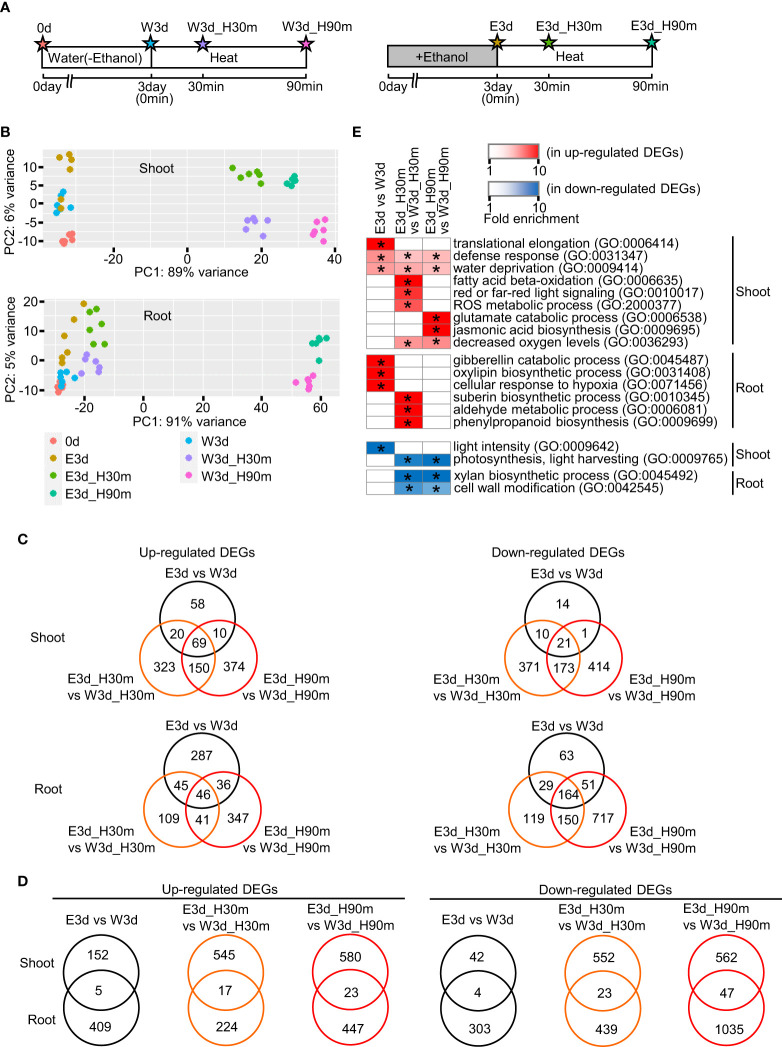
Transcriptome analysis of seedlings heat stressed for 30 min or 90 min. Fifteen-day-old seedlings were pretreated with water or 20 mM ethanol for 3 d then subjected to heat stress. **(A)** Diagrams of sampling time points for transcriptome analysis. Stars show the sampling time points with codes to denote the different treatments. **(B)** Principal component analysis of transcriptome data. **(C)** Venn diagrams of number of differentially expressed genes (DEGs) in comparisons among treatments E3d vs W3d, E3d_H30m vs W3d_H30m, and E3d_H90m vs W3d_H90m. **(D)** Venn diagrams of number of DEGs in comparison between shoot and root. **(E)** Representative GO terms enriched in DEGs. Asterisks show the significance of enrichment (Fisher’s exact test, *P* < 0.05).

To elucidate the temporal and spatial gene expression profiles of ethanol-pretreated and heat stress-treated tomato plants, we performed transcriptome analysis using various organs at different developmental stages. First, we performed RNA-seq analysis using seedlings. **Figure 3A** indicates the diagrams of the sampling time points. Principal component analysis showed that each sample was mostly separated from the others, suggesting different gene expression profiles among these treatments (**Figure 3B**). We then analyzed differentially expressed genes (DEGs) between water- and ethanol-treated samples (**Figure 3**, **Supplementary Figure S6**, **Supplementary Tables S2–S9**). For example, in the shoot, 157, 562 and 603 genes were found as up-regulated DEGs in the comparisons E3d vs W3d, E3d_H30m vs W3d_H30m, and E3d_H90m vs W3d_H90m, respectively. In roots, 414, 241, 470 genes were found as up-regulated DEGs in the same comparisons. Venn diagrams for each DEG set showed that some of the DEGs overlapped among E3d vs W3d, E3d_H30m vs W3d_H30m, and E3d_H90m vs W3d_H90m (**Figure 3C**). For example, among the shoot up-regulated DEGs, 63% (99/157 genes) of the DEGs of E3d vs W3d overlapped with the DEGs of E3d_H30m vs W3d_H30m and/or E3d_H90m vs W3d_H90m. We also compared the DEGs between shoot and root, indicating that most DEGs did not overlap in the comparisons (**Figure 3D**). Gene ontology (GO) analysis showed that various GO terms were enriched in the DEGs (**Supplementary Tables S10–S22**). **Figure 3E** shows the representative enriched GO terms in the up- and down-regulated DEGs. Stress-related GO terms such as “defense response”, “water deprivation”, “ROS metabolic process”, and “jasmonic acid biosynthesis” were found in the up-regulated DEGs while photosynthesis-related GO terms were included in the down-regulated DEGs.

**Figure 4:**
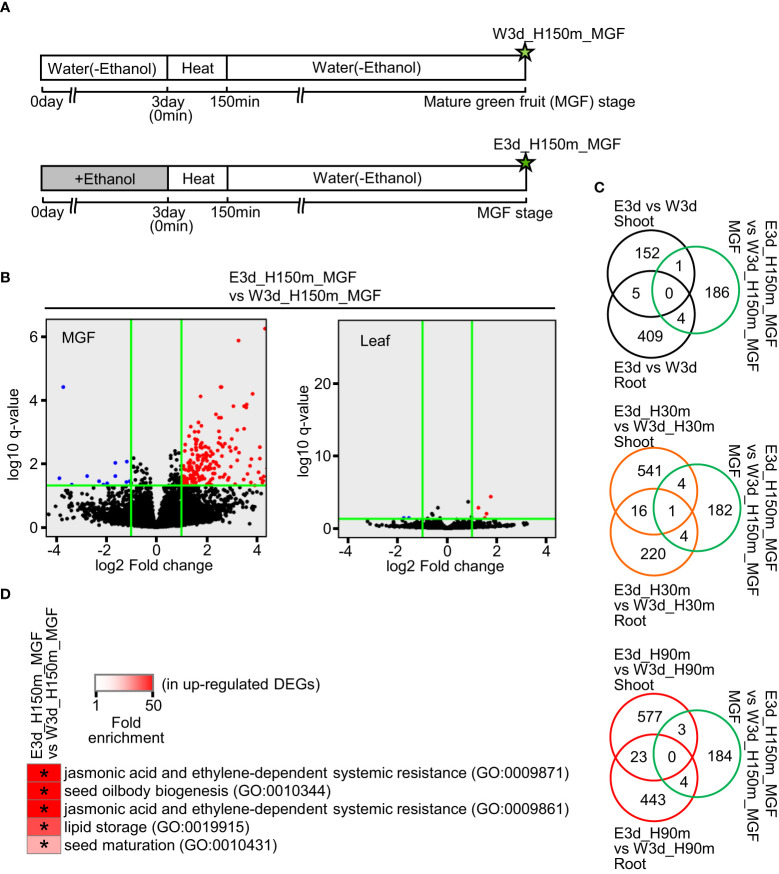
Transcriptome analysis of mature green fruit (MGF) and leaf at MGF stage after water or ethanol pretreatment and heat stress treatment. Fifteen-day-old seedlings were pretreated with water or 20 mM ethanol for 3 d and the seedlings were subjected to heat stress for 2.5 h. After the heat treatment, the plants were grown under normal conditions and MGFs were sampled. **(A)** Diagram of sampling time points for transcriptome analysis. Stars show the sampling time points with codes to denote the different treatments. **(B)** Volcano plots of transcriptome analysis. Horizontal green lines indicate the threshold value of q-value (q < 0.05). Vertical green lines show the threshold values of up-regulated DEG (log_2_ fold change ≥ 1) and down-regulated DEG (log_2_ fold change ≤ 1). **(C)** Venn diagrams of number of DEGs in comparisons as indicated. **(D)** Representative GO terms enriched in up-regulated DEGs in MGF. Asterisks show the significance of enrichment (Fisher’s exact test, *P* < 0.05).

We also performed RNA-seq analysis using samples of mature green fruits (MGFs) and leaves at the developmental stage of MGF after the ethanol pretreatment and heat stress treatment (**Figure 4A**). In the MGF, 191 and 12 genes were identified as up- and down-regulated DEGs, respectively (**Figure 4B**, **Supplementary Table S8**). In the leaf, only four and three genes were identified as up- and down-regulated DEGs, respectively (**Figure 4B**, **Supplementary Table S9**). For further analysis, we focused on the up-regulated DEGs of MGF. Venn diagrams showed that most of the up-regulated DEGs of MGF did not overlap with the DEGs of shoot and root (**Figure 4C**). The up-regulated DEGs of MGF included the enriched GO terms involved in seed development and seed components such as lipids (**Figure 4D**, **Supplementary Table S22**).

To confirm the results of the RNA-seq analysis, we performed RT–qPCR analysis regarding two *SlLEAs* (Solyc02g085150 and Solyc09g014750) and *SlDREB2A* (Solyc05g052410). Similar results were acquired in the RT–qPCR analysis (**Supplementary Figure S7**), which validated the results of the RNA-seq analysis.

### 3.7 Metabolites that were increased or decreased after ethanol pretreatment and heat stress treatment in tomato seedlings and fruits

**Figure 5:**
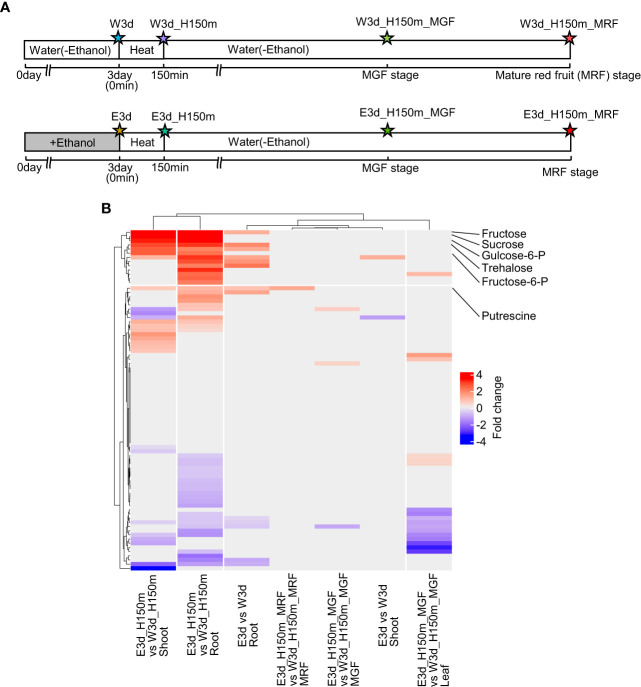
Metabolome analysis. **(A)** Diagram of sampling time points for metabolome analysis. Stars show the sampling time points with codes to denote the different treatments. **(B)** Clustering analysis of fold changes of metabolites between water- and ethanol-treated plants. Only significantly different fold change values (q-value < 0.05) were used in this clustering analysis.

To reveal the metabolites that increased or decreased after ethanol pretreatment and heat stress treatment in tomato seedlings and fruits, we analyzed metabolites by gas chromatography–mass spectrometry (GC–MS). **Figure 5A** shows the time points for this analysis and the codes denoting the different samples. We measured the contents of 81 metabolites (23 amino acids, 9 amines, 18 organic acids, 10 sugars, 3 alcohols, and 18 others) (**Supplementary Tables S23–S29**). Using these data, we identified metabolites differentially accumulated between water- and ethanol-pretreated samples (**Supplementary Tables S30–S37**). In the comparisons of shoot (E3d_H150m vs W3d_H150m) and root (E3d_H150m vs W3d_H150m), it was found that 17 and 25 metabolites were differentially accumulated, respectively (**Supplementary Tables S32, S33**). Clustering analysis showed that some of the ethanol-induced metabolites overlapped between shoot E3d_H150m vs W3d_H150m and root E3d_H150m vs W3d_H150m (**Figure 5B**). They included fructose, sucrose, glucose, fructose-6-phosphate, and glucose-6-phosphate (**Supplementary Tables S32, S33**). The contents of putrescine in root E3d_H150m and mature red fruit (MRF) E3d_H150m_MRF were also higher after ethanol pretreatment than in the water-pretreated samples (**Supplementary Tables S33, S36**).

We also measured the starch contents in the samples at the MGF stage. The contents in ethanol-pretreated fruit and leaf samples were not significantly different from those in water-pretreated ones (**Supplementary Figure S8**).

### 3.8 Enrichment of intrinsically disordered region proteins in up-regulated DEGs

Our previous paper discussed that ethanol treatment might affect the formation of biomolecular condensates driven by liquid–liquid phase separation (LLPS) (Matsui et al., 2022). Because intrinsically disordered region (IDR) proteins have been recognized as an important factor of the LLPS event (Field et al., 2023; Liu et al., 2023), we estimated the enrichment of IDR proteins in the up-regulated DEGs identified by the present RNA-seq analysis. While the proportion of IDR proteins in total tomato proteins was 47.3%, significantly higher proportions of IDR proteins were found in the DEGs of root E3d vs W3d (54.1%), shoot E3dH30m vs W3dH30m (57.1%), and shoot E3dH90m vs W3dH90m (60.7%) (**Table 1**).

**Table 1:** Enrichment of intrinsically disordered region proteins in up-regulated DEGs.

	Up-regulated DEGs	IDR		Ratio (%)
		+	-	
E3d vs W3d (shoot)	157	83	74	52.9
E3d vs W3d (root)	414	224	190	54.1 *
E3dH30m vs W3dH30m (shoot)	562	321	241	57.1 *
E3dH30m vs W3dH30m (root)	241	101	140	41.9
E3dH90m vs W3dH90m (shoot)	603	366	237	60.7 *
E3dH90m vs W3dH90m (root)	470	215	255	45.7
E3dH150m_MGF vs W3dH150m_MGF (MGF)	191	75	116	39.3
Total genes	34074	16132	17942	47.3

*The significance of enrichment was calculated by Fisher's exact test (P < 0.05) as compared to the ratio of total genes in *Solanum lycopersicum*.

## 4 Discussion

The purpose of this paper was to investigate whether ethanol application enhances heat stress tolerance in tomato. As in other plant species reported previously, our results in tomato showed that ethanol pretreatment alleviated heat damage to vegetative growth (**Figure 1**). In addition, we demonstrated that ethanol pretreatment was also effective for improving reproductive development after heat damage (**Figure 2**). Although the fresh weight per fruit was not different between ethanol- and water-treated plants, the fruit number per plant was higher in ethanol-treated plants than in water-treated control plants (**Figure 2**). This suggests that ethanol pretreatment can improve yield of fruits or seeds under heat stress conditions.

Previously, we reported that ethanol pretreatment enhanced heat stress tolerance in Arabidopsis (Matsui et al., 2022). That study showed that ethanol treatment increased the expression level of *Binding Protein 3 (BIP3)*, a marker gene for endoplasmic reticulum (ER) stress (Matsui et al., 2022). We also observed that unfolded protein response (UPR)-related metabolites such as polyamines accumulated in ethanol-treated Arabidopsis plants (Matsui et al., 2022). These results raised the possibility that ethanol treatment might activate UPR signaling in Arabidopsis. This hypothesis was supported by the results that UPR inducer treatment increased heat stress tolerance while UPR inhibitor treatment decreased heat stress tolerance; furthermore, the reduced heat stress tolerance was found in the mutant *bzip60* (Matsui et al., 2022). The protein bZIP60 has been shown to function as an upstream regulator of BIP3 (Humbert et al., 2012; Pastor-Cantizano et al., 2020). In the present study, the expression levels of tomato *BIP* family genes (Solyc01g099660, Solyc01g150132, Solyc03g082920, Solyc06g052050, Solyc08g082820, and Solyc12g055687), *bZIP60* (Solyc10g078290), and also *bZIP28* (Solyc04g082890), which is another upstream regulator of BIP3 (Fragkostefanakis et al., 2015; Löchli et al., 2022), were not different between water- and ethanol-treated samples at seedling stages (**Supplementary Tables S2–S7**). However, it is interesting to note that the content of the polyamine putrescine was higher in some comparisons between ethanol- and water-treated samples (**Figure 5**). Although the expression levels of *BIP3*, *bZIP60* and *bZIP28* were not increased by ethanol application, we found that expression levels of several genes encoding molecular chaperones other than BIP family genes were increased in shoot E3H90m vs W3H90m (Solyc09g092260), in root E3 vs W3 (Solyc05g015470, Solyc07g065970, and Solyc08g005300), and in root E3H90m vs W3H90m (Solyc03g115140, Solyc05g050820, and Solyc08g005300) (**Supplementary Tables S3, S6, S7**). These chaperones might play a role in the UPR machinery of ethanol-treated tomato plants.

Physicochemical properties of ethanol molecules affect liquid–liquid phase separation (LLPS) (Hansen et al., 2021). LLPS has received much attention in biological regulatory processes, not only in yeasts and animals but also in plants (Londoño Vélez et al., 2022; Field et al., 2023; Liu et al., 2023). In the biological aspects of LLPS, accumulating evidence has demonstrated that intrinsically disordered region (IDR) proteins function as important molecules involved in the formation of biomolecular condensates (Han et al., 2023). For example, the yeast prion protein Sup35 functions in the formation of biomolecular condensates to rescue the translation factor from stress-induced injury (Franzmann et al., 2018). We checked the enrichment of IDR proteins in the up-regulated DEGs identified by the RNA-seq analysis (**Table 1**). The proportions of IDR proteins in the up-regulated DEGs (root E3d vs W3d, shoot E3dH30m vs W3dH30m, and shoot E3dH90m vs W3dH90m) were significantly higher than the proportion of IDR proteins among proteins as a whole in tomato (**Table 1**). The observed high proportion of IDR proteins in the up-regulated DEGs suggest that ethanol treatment might induce a cellular state where biomolecular condensates can be easily formed by LLPS. We also found that the GO term “red or far-red light signaling” was enriched in the up-regulated DEGs in shoots (**Figure 3E**). This raises a possibility that phytochrome B regulatory pathways are affected by ethanol application. It is intriguing that phytochrome B functions as one of the thermosensors (Legris et al., 2016; Piskurewicz et al., 2023) and is involved in the formation of the biomolecular condensate photobody in the nucleus (Chen et al., 2022; Shi and Zhong, 2023). Furthermore, we noticed that the expression levels of some of the *late embryogenesis abundant* (*LEA)* genes were up-regulated by ethanol application. For example, the expression level of Solyc02g085150 in the shoot E3d sample was higher than that in the shoot W3d sample (log_2_ fold change = 2.28) (**Supplementary Table S2**). This expression difference was confirmed by quantitative RT–PCR analysis (**Supplementary Figure S7A**). Our RNA-seq analysis showed that 3, 6, and 3 *LEA* genes were up-regulated in shoot E3d vs W3d, shoot E3dH30m vs W3dH30m, and shoot E3dH90m vs W3dH90m, respectively (**Supplementary Tables S2, S4, S6**). LEA proteins function as protective molecules that bind directly to client proteins and prevent aggregation not only under drought stress conditions but also under heat stress conditions (Dirk et al., 2020; Hernández-Sánchez et al., 2022). The shrimp LEA protein AfrLEA6 is involved in desiccation tolerance through LLPS (Belott et al., 2020). If LLPS in the ethanol-treated plants is activated, we suggest that the accumulation of LEA proteins by LLPS might be one of the factors that contribute to enhanced stress tolerance. To elucidate the involvement of ethanol-mediated cellular status in LLPS, further analysis is needed. We are investigating whether ethanol application affects the formation of biomolecular condensates by LLPS.

Bashir et al. (2022) showed that, in ethanol-treated Arabidopsis plants, the ethanol incorporated into cells was converted to sugars via the gluconeogenesis pathway. In our RNA-seq analysis, we found that the expression levels of some genes encoding enzymes associated with ethanol metabolism were up-regulated following ethanol application. The expression levels of the genes (Solyc02g084640, Solyc02g086970, and Solyc05g005700) encoding aldehyde dehydrogenase, which converts aldehyde into acetic acid, were increased in the root E3dH90m (**Supplementary Table S7**). The expression level changes of these genes might reflect the incorporation of the applied ethanol into sugars in the root cells. Because aldehyde molecules are toxic for organisms, the increased expression of genes encoding aldehyde dehydrogenase might be preferred. Our metabolome analysis showed that the contents of sugars, including sucrose, glucose, fructose, glucose-6-phosphate, and fructose-6-phosphate, were increased in ethanol-treated shoots and roots (E3d_H150m) compared with those in water-treated shoots and roots (W3d_H150m) (**Supplementary Tables S32, S33**). This supports the view that ethanol application activates the gluconeogenesis pathway. The GO terms related to photosynthesis were enriched in the down-regulated DEGs (**Figure 3E**), suggesting that sugar accumulation after ethanol application might cause the down-regulation of photosynthesis-related genes and so save photosynthetic effort.

Trehalose is one of the compatible solutes that contribute to modulating osmotic imbalance and stabilizing macromolecules (Dabravolski and Isayenkov, 2022). Our metabolome analysis showed that the contents of trehalose in root E3d, shoot E3d_H150m and root E3d_H150m were higher than those in the water-treated samples (**Supplementary Tables S31–S33**). This suggests that ethanol-pretreated tomato plants might also show improved tolerance to drought stress. The notion was supported by our finding that GO terms such as “water deprivation” were enriched in the DEGs (**Figure 3E**).

In the present study, ethanol pretreatment was applied at the seedling stage. Although the number of fruit was increased by the ethanol pretreatment, the fruit qualitative traits were not greatly altered. We found only one metabolite was increased in MRF (**Supplementary Table S36**). Ethanol treatment at a later developmental stage might alter the dynamics of metabolite accumulation in MRF. Because ethanol pretreatment at the seedling stage increased the sugar contents in seedlings, ethanol treatment at a later developmental stage might cause sugars to accumulate in MRF, giving sweeter fruits. Furthermore, trehalose might also accumulate in the MRF. Because trehalose has therapeutic effects against neurodegenerative diseases in mammals (Yap et al., 2023), trehalose-enriched tomato fruit might prove valuable.

In conclusion, we discovered that ethanol pretreatment alleviated heat-stress-induced damage in tomato, not only during seedling growth but also in fruit development. Transcriptome analysis revealed sets of genes that were differentially expressed in shoots and roots of seedlings and in mature green fruits of ethanol-pretreated plants compared with those in water-treated plants. The sets included genes encoding LEAs and ROS-related enzymes. Metabolome analysis revealed that the contents of some sugars, including trehalose, sucrose and fructose, were increased in the ethanol-pretreated seedlings after heat stress.

**Figure 6:**
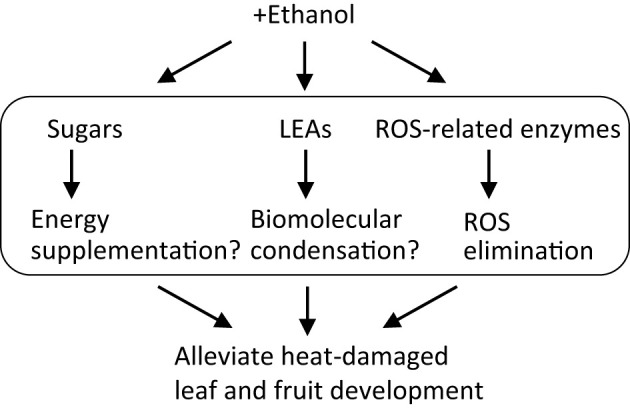
Model for mechanism governing ethanol-mediated heat stress tolerance in tomato.

From these results, we hypothesize a model for ethanol-induced heat stress tolerance mechanisms in tomato (**Figure 6**). Ethanol application increases the contents of sugars, as a result of ethanol incorporation and its conversion into sugars via gluconeogenesis activation. Although environmental stress generally inhibits photosynthesis and growth, in the ethanol-pretreated plants the accumulated sugars improve growth under stress conditions, compensating for reduced photosynthesis. Concurrently, ethanol treatment up-regulates stress-related genes encoding LEAs and ROS-related enzymes. LEAs most likely involve the formation of biomolecular condensation driven by LLPS while ROS-related enzymes decrease the content of toxic ROS molecules. These processes occur cooperatively in the ethanol-treated plants and so increase their heat stress tolerance. The knowledge presented here will encourage the development of ethanol-based chemical priming technology that would reduce heat stress damage and enhance fruit quality in crops, especially in the Solanaceae. At present, regarding this technology, there is a gap on how to apply for agriculture. Research practices in field and horticultural facility using the knowledge presented here will be important for the feasibility.

## Data availability statement

The datasets presented in this study can be found in online repositories. The names of the repository/repositories and accession number(s) can be found below: GEO accession GSE245512.

## Author contributions

DT: Data curation, Investigation, Writing – original draft. DQ: Data curation, Investigation, Writing – review & editing. MT: Formal analysis, Investigation, Writing – review & editing. YU: Formal analysis, Writing – review & editing. CU: Formal analysis, Writing – review & editing. AE: Data curation, Writing – review & editing. ST: Data curation, Writing – review & editing. JI: Formal analysis, Writing – review & editing. MKu: Formal analysis, Writing – review & editing. MKo: Formal analysis, Writing – review & editing. KS: Project administration, Supervision, Writing – review & editing. AN: Data curation, Formal Analysis, Writing – review & editing. YN: Resources, Writing – review & editing. NM: Resources, Writing – review & editing. SF: Resources, Writing – review & editing. MS: Project administration, Supervision, Writing – review & editing.
