# Supplementary material for: Application of ethanol alleviates heat damage to leaf growth and yield in tomato
Source: Front Plant Sci. 2024 Feb 19;15:1325365. doi: 10.3389/fpls.2024.1325365 (PMC10909983; doi:10.3389/fpls.2024.1325365)
Supplement: Supplementary file 1 [file DataSheet_1.zip › Supplementary Figures/Supplemental Figures 2,3,4.pptx]

## Slide 1
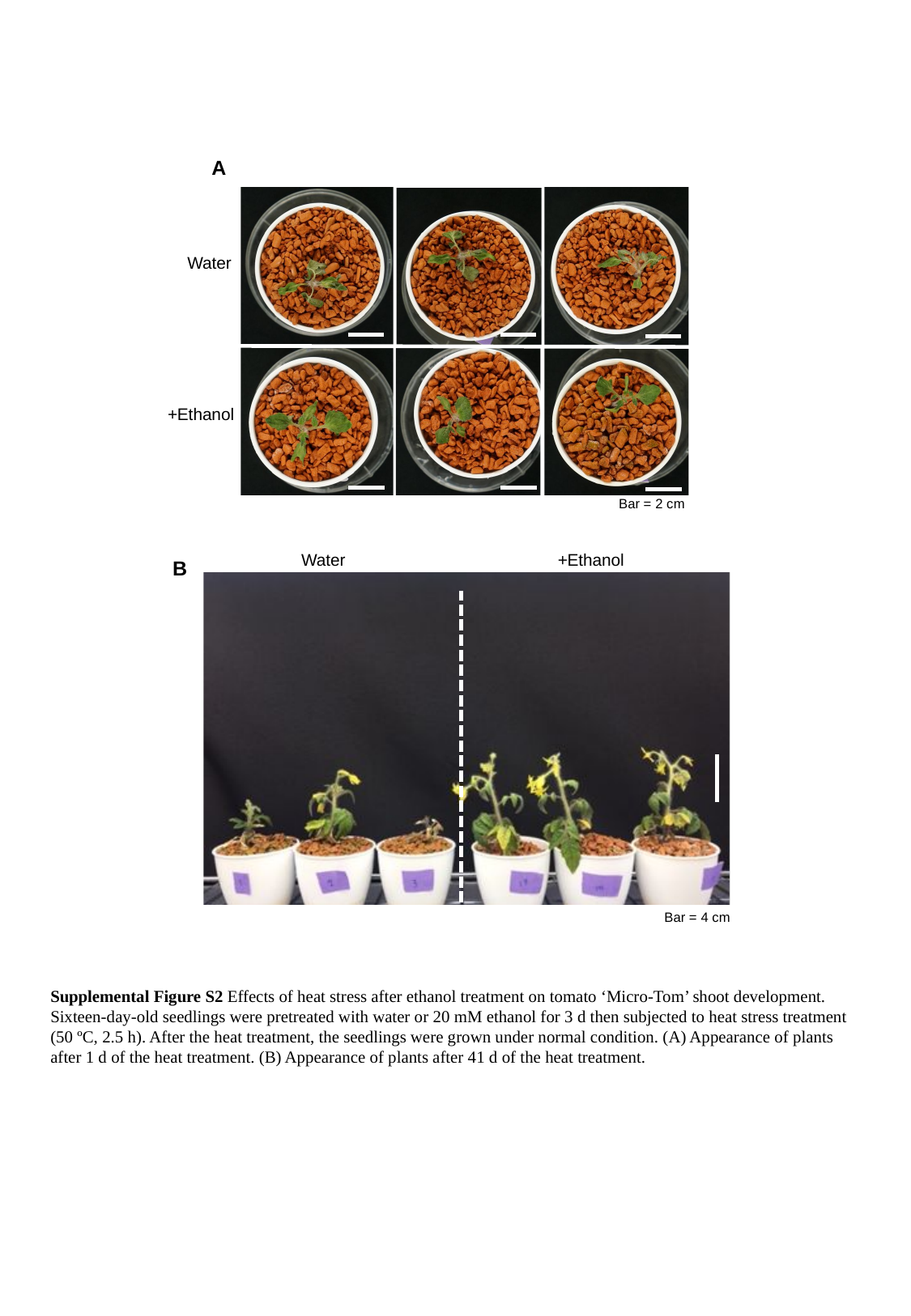

A
Water
+Ethanol
Bar = 2 cm
Water
+Ethanol
B
Bar = 4 cm
Supplemental Figure S2 Effects of heat stress after ethanol treatment on tomato ‘Micro-Tom’ shoot development. Sixteen-day-old seedlings were pretreated with water or 20 mM ethanol for 3 d then subjected to heat stress treatment (50 ºC, 2.5 h). After the heat treatment, the seedlings were grown under normal condition. (A) Appearance of plants after 1 d of the heat treatment. (B) Appearance of plants after 41 d of the heat treatment.

## Slide 2
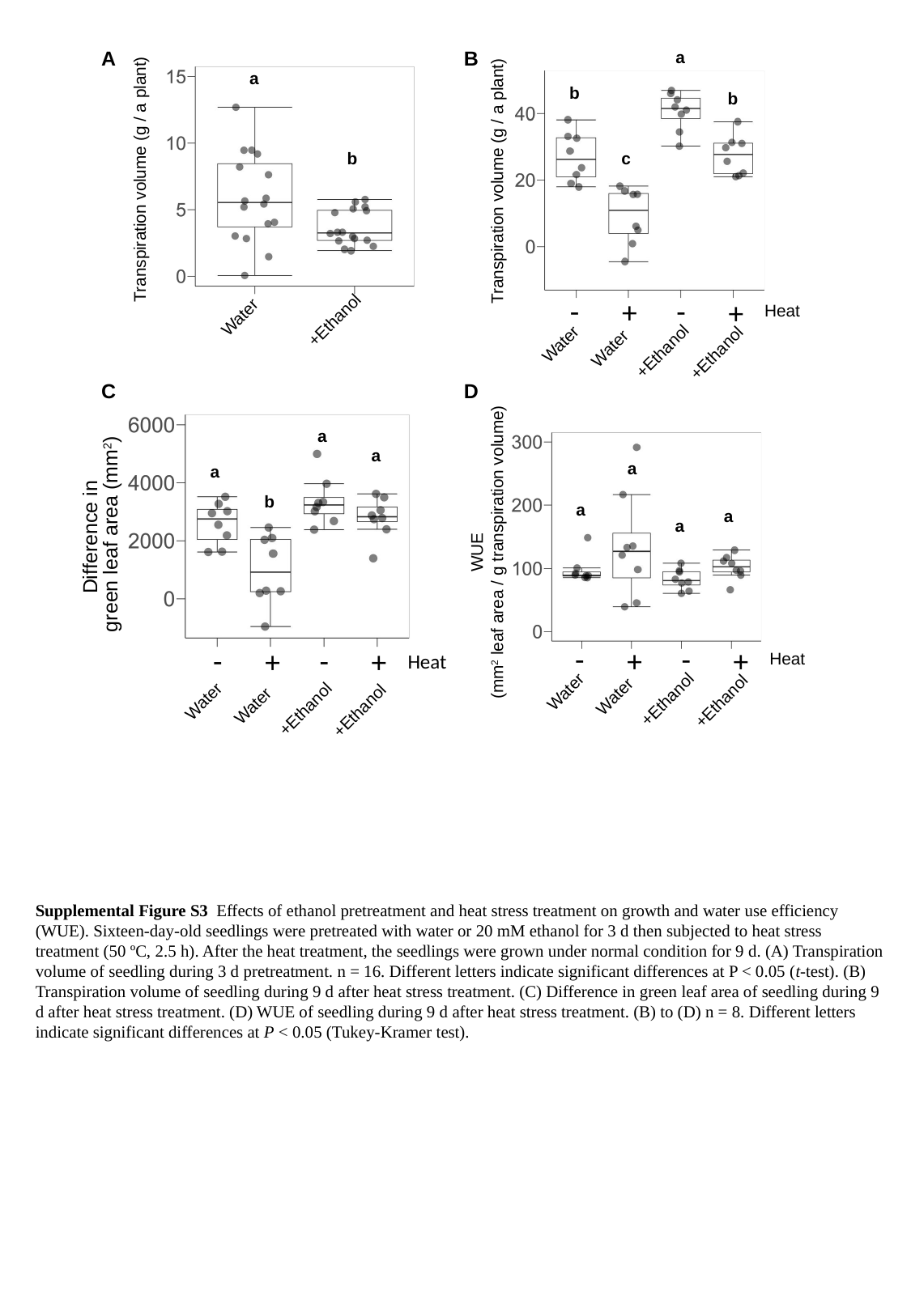

A
B
a
a
b
b
b
c
Transpiration volume (g / a plant)
Transpiration volume (g / a plant)
-
-
+
+
Heat
Water
+Ethanol
Water
Water
+Ethanol
+Ethanol
C
D
a
a
a
a
b
a
a
a
Difference in
 green leaf area (mm2)
WUE
(mm2 leaf area / g transpiration volume)
-
-
+
-
+
-
+
+
Heat
Heat
Water
Water
+Ethanol
+Ethanol
Water
Water
+Ethanol
+Ethanol
Supplemental Figure S3 Effects of ethanol pretreatment and heat stress treatment on growth and water use efficiency (WUE). Sixteen-day-old seedlings were pretreated with water or 20 mM ethanol for 3 d then subjected to heat stress treatment (50 ºC, 2.5 h). After the heat treatment, the seedlings were grown under normal condition for 9 d. (A) Transpiration volume of seedling during 3 d pretreatment. n = 16. Different letters indicate significant differences at P < 0.05 (t-test). (B) Transpiration volume of seedling during 9 d after heat stress treatment. (C) Difference in green leaf area of seedling during 9 d after heat stress treatment. (D) WUE of seedling during 9 d after heat stress treatment. (B) to (D) n = 8. Different letters indicate significant differences at P < 0.05 (Tukey-Kramer test).

## Slide 3
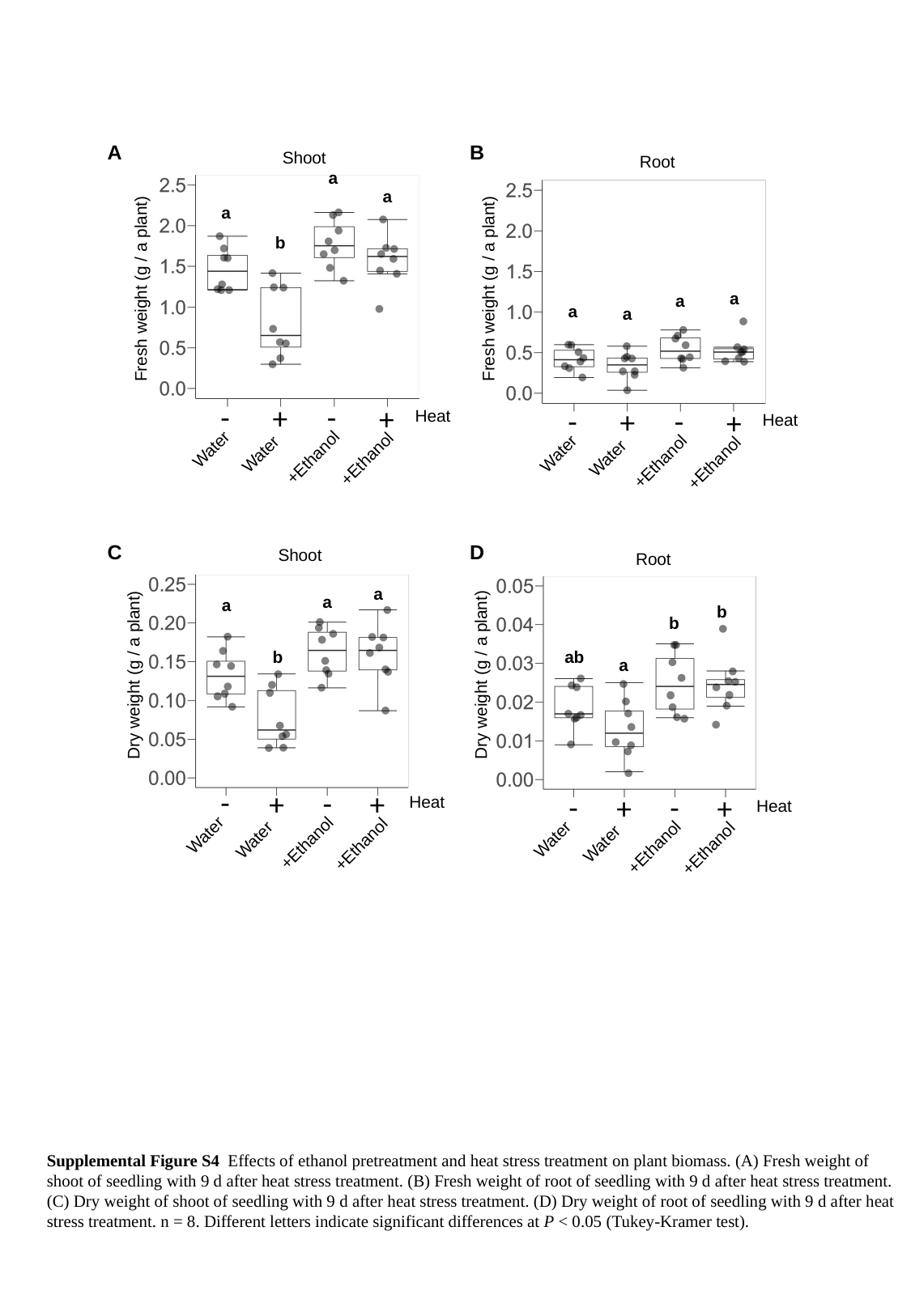

A
B
Shoot
Root
a
a
a
b
Fresh weight (g / a plant)
Fresh weight (g / a plant)
a
a
a
a
-
-
+
+
-
-
+
+
Heat
Heat
Water
Water
Water
+Ethanol
Water
+Ethanol
+Ethanol
+Ethanol
C
D
Shoot
Root
a
a
a
b
b
b
ab
a
Dry weight (g / a plant)
Dry weight (g / a plant)
-
-
+
+
-
-
+
+
Heat
Heat
Water
Water
Water
+Ethanol
Water
+Ethanol
+Ethanol
+Ethanol
Supplemental Figure S4 Effects of ethanol pretreatment and heat stress treatment on plant biomass. (A) Fresh weight of shoot of seedling with 9 d after heat stress treatment. (B) Fresh weight of root of seedling with 9 d after heat stress treatment. (C) Dry weight of shoot of seedling with 9 d after heat stress treatment. (D) Dry weight of root of seedling with 9 d after heat stress treatment. n = 8. Different letters indicate significant differences at P < 0.05 (Tukey-Kramer test).
