# Supplementary figures and images for: Application of ethanol alleviates heat damage to leaf growth and yield in tomato
Source: Front Plant Sci. 2024 Feb 19;15:1325365. doi: 10.3389/fpls.2024.1325365 (PMC10909983; doi:10.3389/fpls.2024.1325365)

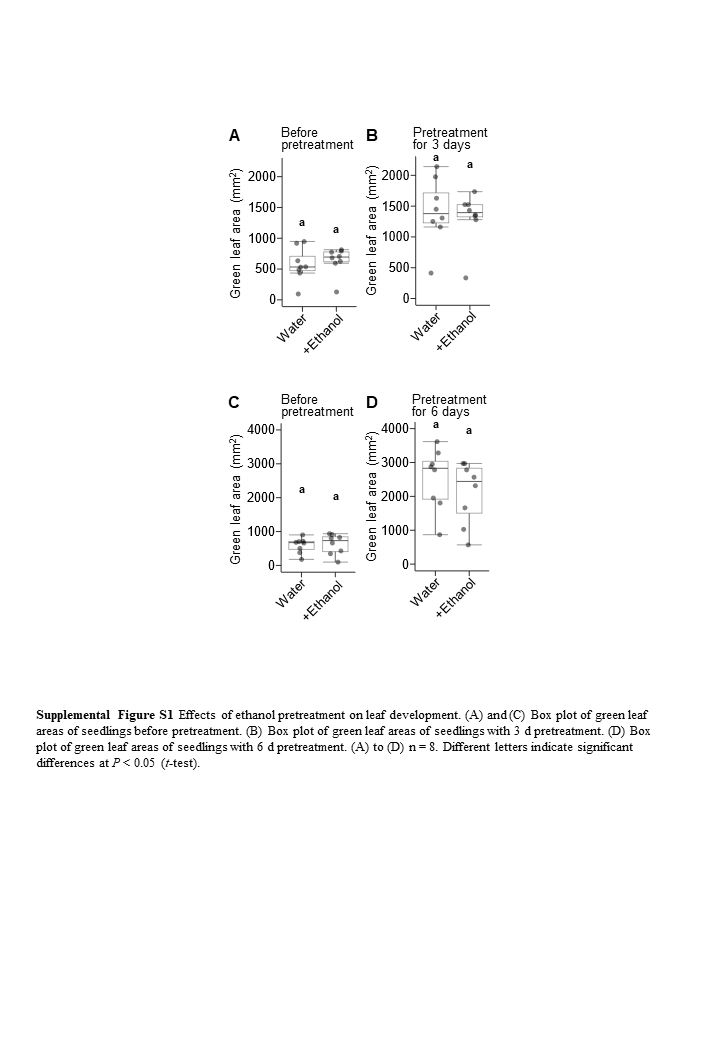

Supplement: Supplementary file 1 [file DataSheet_1.zip › Supplementary Figures/Supplementary Figure 1.TIF]

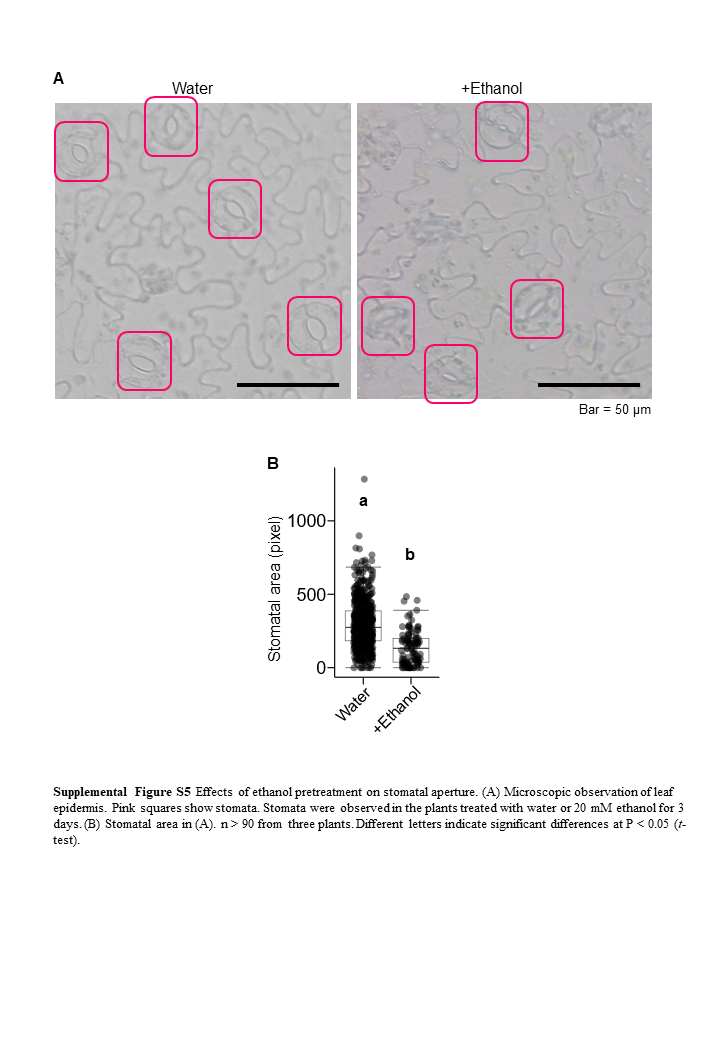

Supplement: Supplementary file 1 [file DataSheet_1.zip › Supplementary Figures/Supplementary Figure 5.TIF]

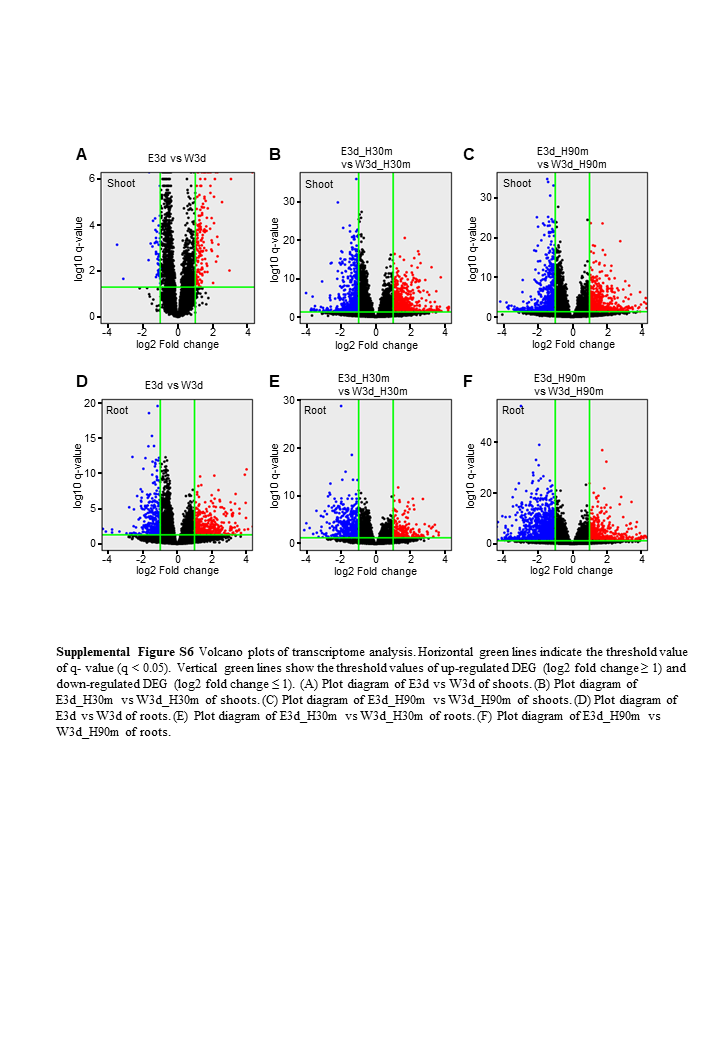

Supplement: Supplementary file 1 [file DataSheet_1.zip › Supplementary Figures/Supplementary Figure 6.TIF]

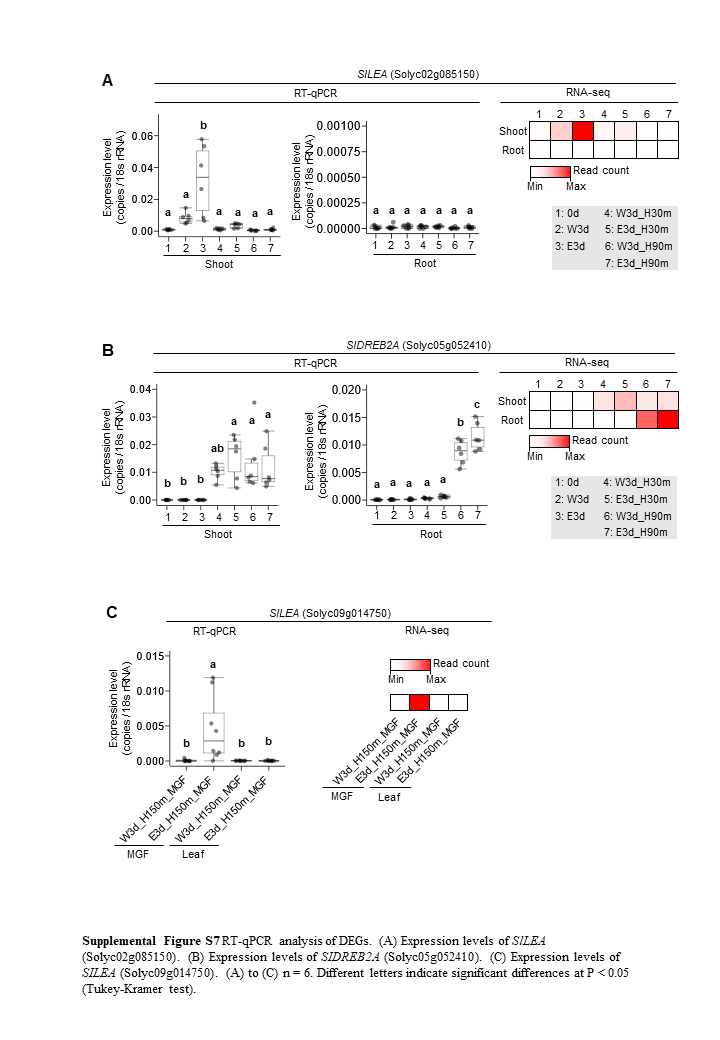

Supplement: Supplementary file 1 [file DataSheet_1.zip › Supplementary Figures/Supplementary Figure 7.TIF]

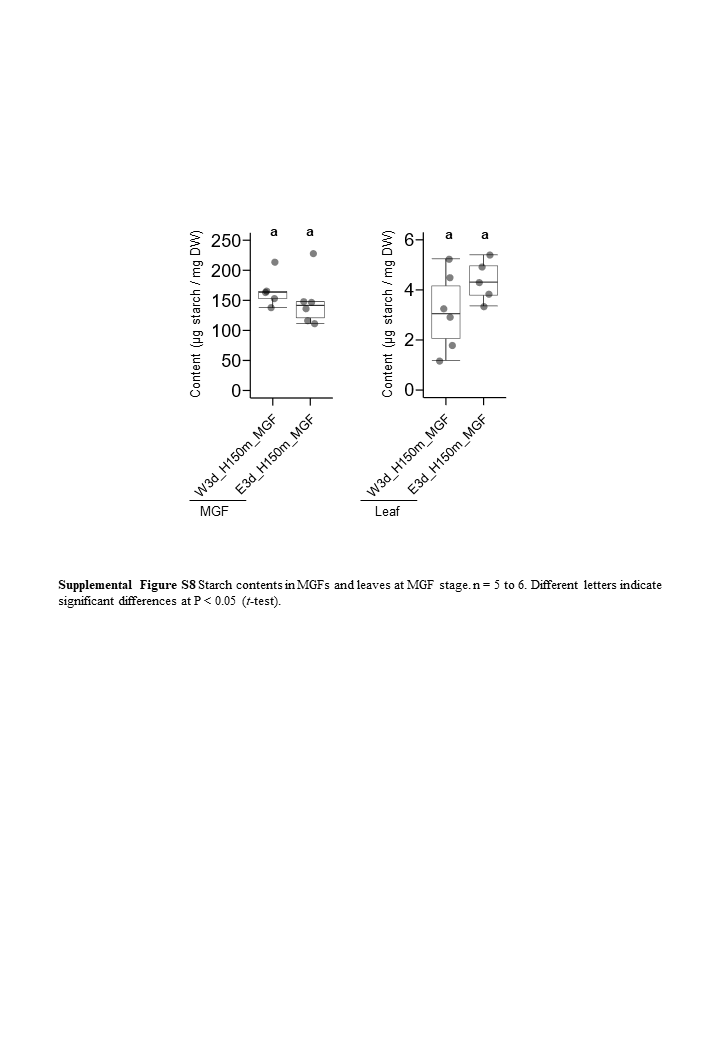

Supplement: Supplementary file 1 [file DataSheet_1.zip › Supplementary Figures/Supplementary Figure 8.TIF]
